# Iodine: Its Actions, Normal and Abnormal, upon the Human Body

**Published:** 1863-07

**Authors:** F. Prakt. Heilk


### IODINE: ITS ACTIONS, NORMAL AND ABNORMAL, UPON THE HUMAN BODY.

BEING A REVIEW UPON THIS SUBJECT, MADE BY DRS. SCHNELLER AND HERMANN, PUBLISHED IN THE OESTRR ZTSCHR. F. PRAKT. HEILK.

*Translated from Canstatt's Jahresbericht.*

Dr. Schneller reports the results of his own experience, which lasted over a period of twenty years; in his practice he employed Iodine, its preparations and certain mineral waters containing it,—his conclusions in reference to its effects being the following: (a) The constitutional effects of Iodine, described by Rilliet under the name of iodismus, offers, in its symptoms, which have been accurately described and grouped together by Rillet, some new features, though its general symptoms have long been recognized as consequences of the use of Iodine. (b) Iodismus, or anglicised *iodism*, only occurs in exceedingly rare cases. (c) Medium, as well as very small doses of the argent give rise to iodism more speedily than larger ones. (d) Old, debilitated persons, and those of an irritable habit, who have manifested symptoms of scrofula, seem to possess, for these reasons, a greater susceptibility to Iodine than others. (e) The use of this agent, in persons of the characteristics just mentioned, requires, on this account, more precaution than has hitherto been exercised. (f) The constancy of the occurrence of iodism as set forth by Rilliet, is not so free from doubt, and hence not of so convincing a character as should induce us to use Iodine less frequently than has hitherto been done.

Somewhat in opposition to these views, it is interesting to note the experience of Dr. Jos. Hermann, which bears much analogy to what has been noticed in Paris and London: Hermann's observations led him to adopt the notion of the non-existence of constitutional symptoms resulting from the use of Iodine. He treated annually, in the department of the Vienna Hospital, devoted to cutaneous and syphilitic diseases, about 1,000 patients. His plan of treatment was as follows: In its incipient stage, syphilis was treated merely as a local affection, without any internal medication; the secondary and tertiary forms of this disease—according to Hermann, sequelæ of mercurial treatment,—were treated entirely by the use of iodide of potassium, iodide of sodium, and iodureted cod-liver oil. Also, as external remedies, were employed the various compounds of Iodine, as well as the tinctura iodini and iodureted glycerine, no mercurials being used: consequently, since more than one-third of the cases had, at the period of their admission, been affected for some time, and been treated with mercury, there were, annually, from three to four hundred patients treated with Iodine. Under this mode of treatment, the following phenomena were present:

(1.) The most ordinary physiological change which took place in the organism was in regard to the urine. This secretion was increased in quantity, and in case metallic poisoning presents itself, the urine is so changed in quantity that its specific gravity sinks to 1005, and even as low as 1002; its solid contents become less in amount, the urea, sulphates, earthy and alkaline phosphates are reduced to amounts so small as to be scarcely appreciable. At the same time the water and the chlorides are increased to abnormal amounts; in this attenuated urine there exist traces of albumen, carbonate of ammonia, and other unusual agents. The augmented urinary secretion, as well as the qualitative changes which occur in it, that have been mentioned, are the most constant phenomena following the use of Iodine,—they occur in near eighty per cent. of the cases; they continue during a variable period, which is influenced by the person's constitution,—the time varying from ten to fifty days, or even more; the increase in quantity disappears, as soon as the urine returns to its normal constitution again; sometimes, also, the supervention of some other symptom, as perspiration, diarrhœa, salivation, occurring under the form of a crisis, is followed by a diminished flow of urine, and a return to its normal constitution. Under the circumstances mentioned, the presence of albumen in the urine is almost a prognostic sign that mercury may be shown by electrolysis.

(2.) In about ten per cent. of the cases in which mercury had been administered, and to an extent which was detrimental to the system, inducing a class of symptoms to which Hermann gave the title of hydrargyrosis, in such cases of syphilis, the administration of Iodine was followed by a profuse secretion of saliva, amounting often to one pound in 24 hours. The salivation thus induced by the use of Iodine is distinguished from mercurial ptyalism in this, that in the former case, there is no orritation or ulceration of the mouth or gums, no swelling of the mucus membrane of the oral cavity, and in the majority of cases, no soreness of the salivary glands, and no unpleasant odor from the mouth;—in many cases, indeed, in which these symptoms had been brought about by the use of mercury, they rapidly disappeared under the use of Iodine. The author, in all such cases, in which salivation has supervened upon the use of Iodine, has found, on examination, that mercury was contained in the salida, and hence he regards this as positive evidence, that the salivation arose from the mercury, and not from the Iodine.

(3.) In about four per cent. of the cases, there followed the administration of Iodine a profuse perspiration, which did not weaken the patient: the author regards this perspiration as critical in character, since it arose under the exclusive use of Iodine alone, no sudorific having been administered; he conjectures that mercury is present in this cutaneous discharge. As was the fact in the case of the occurrence of salivation, so after the supervention of profuse perspiration, the recovery was rapid and permanent.

(4.) In many cases, after the internal use of Iodine, during a period, varying from 20 to 50 days, there appeared an exanthema of the following characteristics: The original form appeared as small, round, papulæ,—in some cases, instead of the papulæ, small vesicles or pustules were present; these were grouped together in patches, disposed at greater or less intervals, which did not become confluent; this eruption, which was attended by no burning nor itching, ran through its various phases in from five to eight days, and then disappeared, without leaving any scar or discoloration. It either presents itself in isolated portions of the body, as on the face, forehead, breast, abdomen, back, or extremities; or it may occur simultaneously in various parts of the body, or, indeed, the entire surface may be affected with it, including even the scalp itself: the general eruption is not accompanied by a universal reaction, but, for the most part, occurs irregularly, so that, in one portion of the body, it has already vanished, while, in other parts, the erosion still shows itself. The color of the spots, on the margin of the vesicles relatively to that of the pustules, is rose-red, in case the mercurial poisoning has been of small extent or is entirely absent; the tinting becomes of a more intense red hue, or eeen of a deep copper color, in case there is a high grade of mercurial poisoning. Also, in tuose instances where the Iodine eruption occurs, in conjunction with some other one, as roseola syphilitica, or with papulæ, pustulæ, or furunculi, which attend mercurial blood-poisoning, then the exanthema, which arises from Iodine,—and which consists of fresh spots or vesicles,—always appears in the intervening interstices, and again disappears,—while the roseola, the papulæ, &c., run through their various modifications. The occurrence of the eruption from Iodine has neither a pathological nor a prognostic significance, and in nowise indicates that the system is surcharged with Iodine and that the agent should be suspended. This phenomenon comes and goes, even during the constant use of Iodine; it appears and disappears during a protracted use of Iodine.

(5.) The scars of chancres, which have long since arisen, even months or years ago, after the use of Iodine, open again; the scars may actually be seen undergoing disintegration and solution of their texture, and thus to present fresh excoriations and even ulcers; these excoriations or ulcers present a great resemblance to the primary chancre, yet do not generate matter which may be inoculated, or propagated by contact. [I would call special attention to this fact, as it is, no doubt, what has fallen under the observation of every one who has treated syphilitic patients with the compounds of Iodine; six weeks since, such an instance came under my notice, in which, during the use of Iodine, the cicatrix of an old chancre opened, presenting all the characteristics of a primary syphilitic ulcer,—and, what was still more remarkable, a bubo soon followed this pseudo-chancre. From my acquaintance with the patient, I was well convinced that he did not deceive me in regard to this chancre occurring sponte sua, as he belonged to that class of men who pride themselves upon the frequency of their venereal attacks—regarding each one as a bright jewel in their life-experience. The bubo, in this case, was caused to disappear by active counter-irritation, without reaching the point of suppuration.—Ed.] The author states that he has seen this event only in the secondary and tertiary forms of syphilis, or, according to his ideas, in the chronic forms of hydrargyrosis. He explains this phenomenon in this wise, that the cicatrices remain dormant so long as the mercurial blood-poisoning is not manifest, or, in case it is manifest, it exhausts its injurious effects in the formation and development of other morbid products;—for example, when it is expended upon the mucous membranes, or upon the glandular or osseous systems. If now the Iodine be given, there arises from the increased activity of the vital process, an augmentation of the vis medicatrix naturæ, under the agency of which there is a disappearance of those cicatrices which have arisen under the employment of mercury:—in order, however, to effect this, the unhealthy scars, which have arisen from the use of mercury, must first be broken down by an ulcerative process, which, after the purification of the blood has been completed, heal up and remain radically cured. Phenomena similar to these have been observed by Dr. Englemann and other physicians, after the use of the iodureted water of the mineral waters of Krueznach.

(6.) In a few cases, perhaps two per cent., there arose, under the use of Iodine, a diarrhœa, which lasted several days, or even some weeks; this diarrhœa, though of a very free character, was unattended with much pain or exhaustion of the body; during its continuance, the external manifestations of hydrargyrosis disappeared before the cessation of the use of the medicine, the diarrhœa also ceased; or, in case the remedy was continued, the diarrhœa in nowise assumed a dangerous character. The author regards this intestinal discharge of a critical character, and especially so when it indicates an elimination of mercury.

(7.) In near one per cent. of the cases in which Iodine was given, the patient's body emitted an unpleasant odor; in other cases, there were objective and subjective indications that the agent had been taken, appreciable in the breath and cutaneous transpiration.

The foregoing phenomena presented themselves in the patient in from eight to ninety days, under the administration of the iodide of potassium, in from 10 to 30 grains, given daily; or, of the pure mineral iodine, in doses of 2 grains daily.—*San Francisco Med. Press.*

